# The Medical Association of Georgia

**Published:** 1896-05

**Authors:** 


### Society Reports.

### THE MEDICAL ASSOCIATION OF GEORGIA.

Abstracts of the Proceedings of the Forty-seventh Annual Meeting, Held at Augusta, April 15, 16, and 17, 1896.

The Association met in the Richmond County Court House and was called to order by the President, Dr. Frank M. Ridley, of LaGrange.

The Address of Welcome was delivered by Dr. Eugene Foster, of Augusta, which was responded to by Dr. E. R. Anthony, of Griffin.

President Ridley then delivered his annual address, in which he forcibly and eloquently dwelt upon several topics of interest to the profession.

The first paper read was by Dr. W. J. Mathews, of Middleton, entitled,

### THE TREATMENT OF PNEUMONIA.

The author had never seen a case in his experience where he thought the symptoms warranted abortive treatment, owing to not seeing the patient until a few hours after the prodromic chill, the lung then being engorged to such an extent that he thought his efforts would prove futile. Having satisfied himself that he has a case of pneumonia to deal with, his first step is to make the patient comfortable. To meet this end he envelopes the affected side with counter-irritation, preferably turpentine. To relieve restlessness, pyrexia and pain he gives phenacetin. Should phenacetin fail he gives morphine hypodermically. To reduce hyperpyrexia he administers phenacetin in sufficient doses every three or four hours in connection with a stimulant, the purpose being to reduce the temperature to 101 or 102 degrees. In his experience, phenacetin does not, like some of the other coal tar products, depress the heart. He advocates the application of blisters. He had never seen a case calling for venesection. To control the pulse he gives veratrum viride every three or four hours with whiskey. The overworked heart should be closely watched, its action sustained, and clot prevented. This he does by digitalis and carbonate of ammonia. In the stage of resolution he resorts to supportive treatment. The type of which he had spoken was the acute lobar variety.

DR. J. W. DUNCAN, of Atlanta, seldom blisters an adult. In the very initial stage a blister might do some good. He does not give the coal tar preparations in pneumonia, for the reason that if they are given to reduce temperature they are liable to produce cyanosis. He frequently uses veratrum viride and considers it a sheet-anchor in this disease.

### COLLES' FRACTURE

Was the title of a paper read by DR. J. B. MORGAN, of Augusta. The most common and frequent fractures with which the general practitioner had to do was a Colles' fracture. The diagnosis of this fracture is not difficult. The history of the accident, the characteristic deformity, pain and seat of the lesion point at once to the nature of the fracture. Every case of Colles' fracture can be readily reduced by strong, forced dorsal flexion. This is best done under an anæsthetic. The best temporary permanent dressing is Wyeth's modification of Pilcher's. The plaster of paris dressing is an excellent one and is to be preferred in old people where there is firm impaction which the surgeon does not care to break up, or in cases where the fragments are more or less comminuted. It should be applied from the lower border of the metacarpus to the middle third of the forearm, with the patient's hand in the straight position. A straight dorsal splint may also be employed, but it is not as desirable as the plaster of paris. Under no circumstances use the angular, crooked or pistol-shaped splint, and no form of splint should be allowed to project beyond the metacarpus. The fingers must remain freely movable to prevent stiffness of the joint. Limited motion should be encouraged at first; later active and free. In aged patients, where we have more or less impaction of the broken ends, reduction should not be attempted as it is better to have a crooked deformed wrist than a failure in bony union and impaction favors the consolidation of the fractured bone.

DR. W. L. CHAMPION, of Atlanta, contributed a paper on

### THE IMPORTANCE OF CAREFUL CHEMICAL AND MICROSCOPICAL EXAMINATION OF URINE IN APPLICANTS FOR LIFE INSURANCE.

Within the last few weeks the author had examined patients with kidney lesions, who a few days or weeks previously had applied for life insurance and had been recommended as good risks. Every medical examiner, after due deliberation, ought to be able, after making a thorough physical examination, having considered the applicant's predisposition to any disease, and last but not least, having made a thorough chemical and, if necessary, microscopical examination of the urine, tell the life expectancy of the applicant before him. Cases were cited by the author to demonstrate that a great many practitioners are too prone to overlook the condition of the kidneys, not to make a careful and thorough examination of the urine, not only in examining applicants for insurance, but their desire to formulate a correct diagnosis in diseased conditions should make it so. He urged the extension of chemical and microscopical investigations in this line of work.

DR. A. W. STIRLING, of Atlanta, in the discussion called attention to some experiments which he had made some years ago, and which formed the basis of a graduation thesis to the University of Edinburgh. Chiefly through the observations of Pavy, Johnson and others it was becoming known that every phase of albuminuria was not a cause of Bright's disease. In order to ascertain the actual number of persons in whom albuminuria was present in health, he examined 369 boys between the ages of twelve and sixteen, who belonged to one of the large training ships on the Thames, near London. In order not to make a mistake in regard to the presence of albumin he examined each one with four different reagents. He found albumin most common three hours after the boys got out of bed and assumed the erect posture when the percentage was by examination 20.8 per cent. Of the boys that played in a band the percentage of albumin was about 60 per cent., while in those that did not play in the band it was only 12.8 per cent. M. Stichel, of Paris, had seen blindness by the playing of wind instruments—due, he says, to passive cerebral-congestion with a similar condition of the retina and choroid.

DR. STIRLING says that the urine is usually found to be absolutely free from albumin while the patient remains in bed, and it makes its appearance within a variable but generally short time after the assumption of the erect posture, and independently of food and all other conditions. The albumin continues in the urine for only an hour or two, or perhaps in varying quantity throughout the day, but as a rule it is disappearing in the afternoon and is entirely gone by bed-time. The ingestion of food has little effect in producing the albumin, and it is not breakfast which causes its frequency of appearance in the morning. Sometimes he had seen a slight rise after a meal, but this is trivial and by no means constant. He had boys brought ashore to the infirmary and confined to bed and then albumin was absent from the urine until they got up, breakfast or other meals having no effect. He had made very many and various experiments in connection with this subject, all tending to prove the same thing.

He examined ninety-two other cases whose ages varied from five to ninety-four with the result that there was an increase in the percentage of people having albuminuria with advancing years. From twenty to thirty years he found the percentage of albumin to be only ten per cent; thirty to forty, twenty-five per cent.; forty to fifty, thirty-six per cent.; fifty to sixty, sixty-six and six-tenths per cent; sixty to seventy, seventy-five per cent.; seventy to eighty, seventy-five per cent.; eighty to ninety, eighty-three per cent. These observations were confirmed by Grainger Stewart.

With regard to the advisability of insuring cases of cyclic albuminuria he would suggest that in the present state of our knowledge the most satisfactory method of dealing would be for the companies to accept an apparently healthy albuminuric at a high premium, agreeing to arrange this on their being satisfied of the disappearance of the albumin for such a length of time as would give reasonable expectation of this being permanent.

### THE MODERN TREATMENT OF SKIN DISEASES.

DR. BERNARD WOLFF, of Atlanta, read a paper on this subject in which he outlined in a succinct manner the progress that has been made in the province of dermatology within the last twenty years. The remedies selected for use in cases of diseases of the skin can be employed in several ways namely, lotions or washes, ointments, pastes, plasters, and soaks, as well as baths. Baths are of two kinds, the vapor and the simple medicated bath made to resemble the natural mineral waters. The chief purpose of the bath is besides cleanliness, to bring about healthy activity of the skin, to remove scales, soften infiltrated areas, increase the elimination of waste products through the skin and to relieve irritable conditions of the skin. Baths are especially valuable in the treatment of parasitic and syphilitic affections of the skin. Ointments and pastes are more frequently used than any other form of preparation in the therapy of skin diseases. The base of an ointment is a matter of importance, for upon it largely depends the benefit to be derived from the medicament. Formerly only lard and wax were used; now we have a greater number of articles to choose from. Of these, vaseline, lanolin, adeps lanae, and resorbin are the most useful. Ledermann has introduced a new excipient called resorbin. It is composed of refined almond oil, emulsified with distilled water with the addition of a small quantity of yellow wax and lanolin. The author's experience with casein ointment leads him to regard it as being incompatible with too many substances to be of much practical value.

The use of soaps is of some importance in the treatment of diseases of the skin. Only neutral soaps, or those containing an excess of unsaponified fat, are to be recommended. Free alkali is destructive to the protective horny layer of the skin. The over-fatty soaps make commendable vehicles for various medicaments, the most frequently used for which are hydronaphthol, resorcin, sulphur, tar, corrosive sublimate. The use of soaps has the effect of encouraging cleanliness on the part of the patient, and cleanliness is one of the prerequisites of successful treatment.

The removal by electrolysis of nævi, moles, warts, and small malignant new growths of the skin is a well established surgical procedure.

DR. M. B. HUTCHINS, of Atlanta, said the modern treatment of skin diseases should embrace internal as well as local or external treatment. The fact that a patient has a constitution should not be overlooked, although if he had to choose between local and internal treatment, he should prefer the former in the treatment of ninety-nine per cent. of the cases of affections of the skin. He had not given the plaster mulls of Unna much trial. They had been objected to on account of containing a gutta-percha base, and in any event the dermatologist would have to carefully select the cases in which to use them. With reference to soaps, such as the bichloride of mercury, the chemical change which takes place renders the remedy inert, and it is almost impossible to get any absorption through the unbroken epidermis. Success in the treatment of syphilis by mercurial inunctions is attained, not by the absorption of the mercury through the skin, but by inhalation of the vapors from the mercurial ointment.

As regards gelatin paste he had used it a little, but thought it was objectionable because it stuck to everything with which it came in contact, particularly woolen goods.

In some cases of acne on the faces of young ladies he had obtained beneficial results by using the constant galvanic current to the cheeks where the eruption appeared. The stimulating action which takes place encourages the glands to throw off secretions without allowing them to remain sluggish and thus block up the follicles of the skin.

### APPENDICITIS.

A paper on this subject was read by DR. SAMUEL LLOYD, of New York City. It was largely statistical. In making his compilation the author began with the very earliest recorded cases, and it covers a greater number of medical than surgical cases. The total number examined up to April 5, 1896, was 558, and the result was that 263 recovered and 295 died. Of this number 226 were operated upon, 192 recovered, and 31 died. On the other hand, in direct contrast to this, 265 cases were treated conservatively, 60 recovered, while 205 died. A case was cited which the author had seen in consultation, illustrating the course of extra-peritoneal abscesses.

According to the table of cases that the author had examined, the pathological conditions could be summed up as follows:

1. Cases in which the disease follows traumatism.

2. Cases following septic inflammations in other parts of the body, such as salpingitis in the female, post-partum septic inflammation of the uterus, typhoid fever, tuberculosis.

3. Direct infection of the mucous membrane of the appendix by its contained bacteria.

4. Alterations in its position and shape so as to occlude its lumen, preventing the escape of its natural secretions and contained intestinal contents.

5. Changes in its position and pressure upon its mesentery by growths, or impacted feces in the cæcum.

6. Alterations in its position or shape so as to shut off its blood supply.

7. Foreign bodies, including fecal concretions.

8. There was one other pathological condition about which the author could obtain no information from the cases tabulated, namely, the abdominal tonsil, referred to by Sutherland, Robinson, Yeo Burney, Haigh, and Bland Sutton.

It is probable that in the present state of surgical knowledge the majority of medical men are in favor of surgical intervention, in cases tending toward general peritonitis, even though the symptoms do not point to perforation, or abscess formation, in the hope that drainage of the abdominal cavity and the removal of the focus of disease may enable the surgeon to forestall what would otherwise be a fatal complication.

Of 445 cases out of 558 that were examined, 79 per cent. resulted in abscess, perforation, peritonitis, etc.

The following table gives the full data:

| | |
|---|---|
| Perforation, without given cause | 11 |
| With abscess | 10 |
| With ulceration | 16 |
| With peritonitis | 32 |
| With concretion of foreign body | 41 |
| With gangrene | 7 |
| With gangrene and concretion | 19 |
| With inflammation | 13 |
| With hardened feces | 1 |
| With concretion and peritonitis | 24 |
| With foreign body and peritonitis | 7 |
| | 181 |

ABSCESS WITHOUT PERFORATION.

| | |
|---|---|
| Uncomplicated | 136 |
| Containing concretion | 10 |
| With perforation | 2 |
| Sloughing appendix | 2 |
| Foreign Body | 1 |
| | 160 |
| Foreign bodies | 36 |
| Fecal concretions | 26 |
| Inflammation of the appendix | 8 |
| Enteroliths | 2 |
| Gangrene of the appendix | 9 |
| Gangrenous appendix with concretion | 2 |
| Inflammation with concretion | 3 |
| | 86 |

GENERAL PERITONITIS.

| | |
|---|---|
| With concretion | 5 |
| Foreign body and gangrenous appendix | 1 |
| Foreign body | 3 |
| Ulceration | 1 |
| | 10 |
| Total | 437 |

It will be noticed that this is a dismal showing for the conservative treatment of appendicitis. The author believes that no case of appendicitis should ever be considered as purely medical. When one is called to a patient it is his duty to immediately prepare for operative interference. He would advise operation in every case, after studying the subject carefully, where the symptoms showed any tendency to increase. If a tumor was present and the symptoms suggested the possibility of the presence of pus, he would surely operate.

DR. E. H. RICHARDSON, of Atlanta, followed with a paper on THE MEDICAL SIDE OF APPENDICITIS. He cited the opinions of eminent practitioners, both in this country and Europe, in favor of non-operative interference in a large proportion of cases.

DR. W. H. ELLIOTT, of Savannah, said the foremost difficulty in cases of appendicitis was to know when and when not to cut. No man was fit to treat appendicitis who was not absolutely willing at any moment that he might be needed to open the abdominal cavity.

DR. WILLIS F. WESTMORELAND, of Atlanta, was quite sure that in every case of relapsing appendicitis he had seen there were adhesions from previous attacks. Some of these cases had died. If they had been operated on in the primary attack, the chances would have been better for their recovery. He would open the abdomen as soon as appendicitis was diagnosed if the patient would permit it.

DR. JAMES A. WRIGHT, of Augusta, considered the operation for this disease very difficult, for the reason that no two cases were alike. It was an easy matter to cut down, but if pus and adhesions were found around the appendix the operation was extremely difficult.

DR. J. W. DUNCAN, of Atlanta, has seen and treated medicinally several cases of appendicitis during 30 years' practice, one of them having died from a recurrent attack a few months after the primary one. He was satisfied that many of the cases operated upon were nothing but typhoid fever, and that other cases thought to be typhoid fever were really appendicitis.

DR. J. B. S. HOLMES, of Atlanta, considers the disease a surgical one, and thinks it is safer to operate as soon as a diagnosis is made. The only cases he had lost were those that had been treated medicinally from two to four days previous to operative interference. If an early operation is performed, the mortality should be small.

DR. WM. H. DOUGHTY, of Augusta, maintainedthat it was not prudent to operate upon every case of the disease as soon as the diagnosis was made, for the reason that the surgeon does not always see the cases in their incipiency. If he did, it would be safe to remove the appendix as the chances were that at this time there would not be pus formation; in other words, the surgeon would have a comparatively clean condition within the abdominal cavity and the mortality of operations done under these circumstances ought to be small.

DR. M. L. BOYD, of Savannah, took the position that if all cases could be diagnosed early, it would be wise to operate at once.

DR. GEORGE H. NOBLE, of Atlanta, thought it was extremely unfortunate that the general practitioner should view appendicitis from a prejudiced standpoint. There was undoubtedly many cases of the catarrhal form that were relieved temporarily, or cured by purgation, possibly permanently. Where we have rupture and the discharge of the contents of the appendix into the peritoneal cavity, we have the fulminant form of the disease, and in such cases, very prompt and active measures must be instituted. He advocated early operation.

DR. L. G. HARDMAN, of Harmony Grove, said that while there were cases of appendicitis calling for the services of the surgeon, there were others that could be treated successfully by medicinal measures. Of 15 cases which had come under his care, only 2 were operated upon. All recovered, both the operative and non-operative cases. In the cases operated on suppuration had occurred, and one of the patients, a physician, was present.

### SOME ALBUMINURIC COMPLICATIONS OF PREGNANCY.

A paper on this subject was read by DR. HOWARD J. WILLIAMS, of Macon. He said this complication was present in from 6 to 50 per cent. of all pregnancies. On the other hand puerperal eclampsia was comparatively rare. From January 1st, 1891, to March 31st, 1896, he had encountered 10 cases of albuminuria or toxæmia in 163 pregnancies, and 2 cases of eclampsia in this number—10 cases. This gives 6 per cent. of toxæmia and about 1 per cent. for eclampsia. The percentage given is based upon accurate analysis of the urine of every woman he had attended during the time mentioned. Albuminuria may be present in varying degrees, from a mere trace to a very large per cent., the urea and other excreta accumulating in the blood in the same proportion. The toxæmia is slow or rapid, according to the degree of retention of the latter from the outside. These variations were shown by the recital of three cases.

The cardinal indications are to promote elimination of the toxic materials circulating in the blood and to restore the excretory organs to their normal functions. If, however the poisoning is excessive and the life of the mother and the fœtus is at stake, then the indication is to promptly empty the uterus.

Elimination is pre-eminently indicated for all forms of kidney disease, and in this complication it yields the best results. But if it cannot be tolerated, or disagrees, a more liberal diet should be given, such as the white meats of fowls, fish, oysters, fresh fruits, and good nutritious breads. Remedies to promote elimination by the kidneys were then dwelt upon. Nervous excitement should be controlled by sedatives and narcotics, though not to the extent of interfering with eliminative measures. Elimination of the waste products having failed to restore the functions of the excretory organs and the condition is urgent, eclampsia being imminent or other complications alarming, the uterus should then be emptied.

DR. R. B. BARRON, of Macon, delivered the ORATOR'S ADDRESS. He dwelt upon and pleaded for higher medical education. Relative to practicing medicine for money, he said that money with the true physician was of secondary consideration; that the real incentive for the best work with those practitioners who achieve success was that broad humanitarianism which impelled with resistless force the commissioned agents of God Almighty to relieve suffering, to stay the pangs of agonizing pain, to fight to the bitter end humanity's implacable and unconquerable enemy—death—regardless of any other consideration.

### THE AFTER-TREATMENT OF TRACHEOTOMY CASES OF MEMBRANOUS CROUP.

was the title of a paper read by DR. R. M. HARBIN, of Rome, in which he drew the following conclusions:

1. Croup, whether diphtheritic or membranous, is almost invariably fatal without surgical treatment, and the few cases that recover by medicinal treatment alone are not to be considered.

2. So far as the practical indications for tracheotomy are concerned, it makes no difference whether croup be diphtheritic or membranous.

3. Tracheotomy has the advantage over intubation, in that it gives a better means of expectorating the membranes and furnishes free drainage from the site of septic infection.

4. Tracheotomy is a justifiable surgical procedure, and should be performed in all cases where our therapeutic re-sources have been exhausted, and where the patient is in imminent danger of suffocation. It should be done in hopeless cases, since it either offers a chance for the patient or promotes euthanasia.

5. Tracheotomy keeps the patient alive until the pseudo-membrane disintegrates and resolves into a muco-purulent liquid and is expectorated through the tube.

6. The after-treatment is the most important part of the procedure and the author attributes the successful results reported to the persistent use of lime-water.

DR. J. B. S. HOLMES, of Atlanta, reported several interesting surgical cases.

### A CASE OF ENORMOUS VENTRAL HERNIA CURED BY A PLASTIC OR FLAP OPERATION.

By DR. GEORGE H. NOBLE, of Atlanta. The subject was a very large woman who came to him from a neighboring state, giving a meagre history, saying that the protrusion first appeared after severe straining, and grew rapidly until it reached the size of an adult head. The treatment she had received consisted in local applications only, no attempt at operative measures having been made. The case is of considerable interest on account of such a large hernia in this region and because the expansion of the ribs prevented closure of the ring by approximation of its margins, necessitating therefore a plastic or flap operation to close the aperture, which was large enough to pass a closed hand through without resistance.

The operation consisted of (1) in trimming away the excess of the sack and uniting the peritoneum with buried catgut sutures. (2) Four strong tension sutures were passed through the abdominal walls, piercing the semilunar lines down to the peritoneum, but not implicating it. (3) The semilunar flap was carefully outlined upon either side over the recti muscles with a straight or vertical side upon their outer margins and the convex borders turned toward and extending to the hernial ring. The aponeurosis of the external oblique and the outer layer of that belonging to the internal oblique muscles were cut through and the flaps liberated except where their bases joined the ring and turned over the opening, accurately abutting the edges, in which position they were stitched with buried silk sutures. The convex borders coincided with the margins of the ring to which they were made fast. The recti muscles were brought in direct apposition by surrounding them with the large catgut, thus adding another layer of strong tissue over the hernial opening. (5) The skin and fatty tissue were then brought together and the tension sutures tied over all, the wound dressed antiseptically, with firm pad, roller bandages, etc. The wound proved entirely aseptic, and the result was very gratifying.

In another such case the reader would use buried silver sutures instead of absorbable materials.

DR. M. B. HUTCHINS, of Atlanta, read a paper on the

TREATMENT OF SKIN DISFIGUREMENTS BY ELECTROLYSIS.

That electrolysis has a wide application and a legitimate use, he was convinced by his own experience during the past five or six years. To do the work outlined in the paper a few galvanic cells, a sponge electrode, a needle-holder, and for accurate work, a milliamperemeter, are necessary. In most cases the needle should be attached to the negative pole. The current used is weak, and varies from 3 to 7 or 8 cells. With a milliamperemeter it is found that we get from 1 to 5 milliamperes from this current to the relative position of the electrodes, the greater amount of intervening tissue reducing the current by resistance, a lesser interval increasing it. The number of needles used, the manner of grasping the sponge electrode, the wetness of the sponge, all influence the strength of the current. The strength of current used in these operations is harmless to the patient and would not be felt upon the unbroken epidermis.

For the removal of superfluous hairs on the faces of ladies, electrolysis was the only method. Many of the patent hair-removers were excellent stimulants to its growth. A lounge, good eyes, a good light, a steady hand and perseverance were necessary. Dr. Hutchins had never seen any injury result from the treatment of moles by electrolysis. He had removed one by excision, which had become epitheliomatous through a razor-cut. There was no recurrence. Moles with hairs in them will frequently disappear from the simple effect of the current used in destroying the hairs, but destroying the mole does not always destroy its hairs, as they are deeper.

Warts of various kinds, so tedious of treatment with the old methods, save that of excision, with or without cauterization, usually dissolve into froth under the action of electrolysis, being more easily destroyed than the average mole. The author then dwelt upon electrolysis in the treatment of epithelioma, small fibromata, milia, sebaceous cysts, keloids and hypertrophic scars, comedones, angiokeratoma, etc.

The paper was based almost entirely upon the author's own practical experience, and his implicit faith in this method of treatment is due both to results in practice and upon himself.

DR. M. L. BOYD, of Savannah, followed with a report of a few interesting surgical and gynæcological cases.

### THE USE OF SUBCONJUNCTIVAL INJECTIONS OF MERCURY IN CERTAIN FORMS OF EYE DISEASES.

By DR. DUNBAR ROY, of Atlanta. In this paper the author tabulated twenty-five cases of various eye affections, and his experience leads him to the following conclusions: (1) In infected processes of the cornea, ulcers, abscesses, wounds, etc., this method of treatment is quicker, and therefore more satisfactory for clinical purposes, than those usually employed. (2) In acute iritis it does not seem to exert any beneficial influence, but produces great pain, while in other cases the pain is mitigated. (3) In chronic iritis and iridocyclitis the pain and congestoin are often markedly influenced for the better, but it has no effect whatever upon adhesions of the iris to the lens of the capsule. (4) In post-operative infection and panophthalnit it is by far the best method to which we may resort for good results. (5) In pannus of the cornea absolutely no effect was produced one way or the other. (6) With lesions of the choroid and other deeper structures of the eye he had had no experience, but expects to try this method whenever a case presents itself. From his personal experience he would unhesitatingly say that we can depend upon subconjunctival treatment alone, but as an adjuvant it is most excellent, and in some cases produces results far more beneficial than were expected.

DR. A. W. STIRLING, of Atlanta, read a paper on

### GLAUCOMA IN RELATION TO GENERAL PRACTICE.

He said that glaucoma was to the ophthalmic specialist one of the most interesting diseases, comprising 1 per cent. of all-ophthalmic troubles. While he believes that every case of the disease requires the most skillful advice that can be obtained, still it is the first and foremost affection which comes within the province of the general practitioner, and with him lies frequently the ultimate safety or destruction of the eye involved. The reader reported several interesting cases, after which he dwelt upon the variability in tension. Coming to the treatment he counseled against the indiscriminate use of mydriatics, especially atropine, scopolamin, and cocaine, in affections of the eye. Glaucoma had often resulted from the use of these agents and is almost always accentuated by them. In most ocular diseases they are harmful, and the general practitioner would do well to remove them from his lists in ophthalmic practice, except when he knows he is dealing with an uncomplicated keratitis or inflammation of the iris. On the other hand, in myotics, and notably in eserine and pilocarpine, we have a fairly certain means of temporarily benefiting many cases of glaucoma and of saving valuable time till the most suitable method of treatment can be decided on.

The following officers were elected:

President—Dr. Geo. H. Noble, Atlanta.

First Vice-President—Dr. J. B. Morgan, Augusta.

Second Vice-President—Dr. R. B. Barron, Macon.

Secretary—Dr. R. H. Taylor, Griffin.

Treasurer—Dr. E. C. Goodrich, Augusta.

Place of meeting—Macon, 1897.

